# Non-ST-Segment Elevation Myocardial Infarction (NSTEMI) in a High-Risk Rheumatoid Arthritis Patient on Long-Term Baricitinib: Drug Signal or Risk Factor Convergence?

**DOI:** 10.7759/cureus.109913

**Published:** 2026-05-30

**Authors:** Ali D Alghaithi, Khalid A Alnaqbi

**Affiliations:** 1 Internal Medicine, Sheikh Tahnoon Bin Mohammed Medical City, SEHA/PureHealth, Al Ain, ARE; 2 Rheumatology, Sheikh Tahnoon Bin Mohammed Medical City, SEHA/PureHealth, Al Ain, ARE; 3 Internal Medicine, College of Medicine and Health Sciences, United Arab Emirates University, Al Ain, ARE; 4 Medicine, RAK Medical and Health Sciences University, Ras Al Khaimah, ARE

**Keywords:** aged, baricitinib, janus kinase inhibitors, myocardial infarction, rheumatoid arthritis

## Abstract

Rheumatoid arthritis (RA) is associated with increased cardiovascular (CV) morbidity and mortality, partly related to chronic systemic inflammation and accelerated atherosclerosis. Janus kinase (JAK) inhibitors, including baricitinib, are effective targeted therapies for RA, but their use in older patients and those with baseline CV risk factors requires careful risk assessment. We report a clinically detailed case of non-ST-elevation myocardial infarction (NSTEMI) during long-term baricitinib therapy and use it to discuss real-world CV risk stratification, monitoring, and pharmacovigilance in patients with RA receiving JAK inhibitors.

A 77-year-old Omani woman with seropositive erosive RA since age 69 (Month 0), poorly controlled type 2 diabetes mellitus, well-controlled hypertension, well-controlled hyperlipidemia, obesity, and family history of myocardial infarction was treated with baricitinib 4 mg daily (Month 47) after inadequate response to previous disease-modifying antirheumatic drugs. Her RA remained in sustained remission for approximately 33 months on baricitinib and methotrexate. A cardiac evaluation performed five years earlier, including echocardiography and dobutamine stress testing, had not shown evidence of inducible ischemia or structural heart disease at that time. She subsequently developed sudden chest pain during physiotherapy, was diagnosed with NSTEMI (Month 79), and underwent percutaneous coronary intervention for severe calcified coronary artery disease with critical left anterior descending and diagonal branch stenoses. Baricitinib was discontinued at Month 80, and she remained in RA remission on methotrexate alone without recurrent CV events through her most recent follow-up (Month 108).

A comprehensive literature review using MEDLINE/PubMed, Scopus, EMBASE, and Google Scholar identified only three relevant cases describing NSTEMI during baricitinib therapy in RA, but they lacked sufficient clinical detail for meaningful comparison. Naranjo adverse drug reaction probability scale supports a possible association. This case highlights the need for structured CV risk reassessment, shared decision-making, and pharmacovigilance during long-term JAK inhibitor therapy in high-risk patients with RA.

## Introduction

Cardiovascular (CV) comorbidities are highly prevalent in rheumatic diseases, driven by both traditional risk factors, such as age, diabetes, hypertension, hyperlipidemia, obesity, and smoking, and immune-mediated mechanisms that promote endothelial dysfunction and accelerated atherosclerosis [1]. Rheumatoid arthritis (RA) is a chronic systemic autoimmune inflammatory disease primarily affecting the joints and is independently associated with an increased risk of CV disease compared with the general population [2], partly through persistent systemic inflammation that promotes accelerated atherosclerosis via endothelial dysfunction and immune-mediated vascular injury [1,3].

Baricitinib is an oral Janus kinase (JAK)1/2 inhibitor and a targeted synthetic disease-modifying antirheumatic drug (DMARD) which was approved in the European Union in February 2017 [4] and by the US FDA in 2018 [5] for use as monotherapy or in combination with methotrexate in adults with moderate to severe RA who have responded inadequately to one or more DMARDs or are intolerant to them. Baricitinib is effective in controlling inflammatory disease activity; however, CV safety concerns around JAK inhibitors have led to greater caution, particularly in older patients and those with baseline CV risk factors. The precise mechanism remains unclear, but any potential contribution of JAK1/2 inhibition to CV events is likely to be context-dependent and influenced by baseline risk, lipid changes, thrombo-inflammatory pathways, and underlying atherosclerotic disease [6].

Existing evidence on baricitinib-associated CV risk remains conflicting and difficult to interpret. Randomized controlled trials (RCTs) and long-term extension studies provide important safety data but may under-represent elderly, multimorbid patients with high baseline CV risk. Pharmacovigilance databases can identify potential safety signals, but they often lack detailed clinical context and cannot establish incidence or causality. Real-world case-based evidence is limited, especially in Arab countries [7,8]. Therefore, clinically detailed real-world cases may add value by illustrating how baseline CV risk, RA-related inflammatory risk, treatment exposure, patient preference, and evolving safety concerns converge in routine practice.

We present a detailed case of non-ST-elevation myocardial infarction (NSTEMI) occurring during long-term baricitinib therapy in a high-risk elderly patient with RA, incorporating longitudinal rheumatology follow-up, CV risk assessment, association evaluation, and the patient’s perspective. The purpose of this report is not to establish baricitinib as the cause of NSTEMI, but to describe a temporally associated event in the context of competing CV risk factors and to highlight practical issues in CV risk stratification, periodic reassessment, shared decision-making, and pharmacovigilance during long-term JAK inhibitor therapy.

## Case presentation

A 77-year-old Omani woman had seropositive erosive RA since age 69 (Month 0), total knee replacement bilaterally at age 68, poorly controlled type 2 diabetes, well-controlled hypertension, well-controlled hyperlipidemia, obesity, and several traumatic fractures (left elbow at approximately age 51, left rib and right shoulder at approximately age 61, left foot at approximately age 65, and right toe at approximately age 73). She was a lifelong non-smoker and never consumed alcohol. Her sister had a myocardial infarction (MI) in her 60s, and her nephew died of MI in his 40s.

Her RA was diagnosed at age 69 (Month 0) based on polyarthritis and highly positive anti-cyclic citrullinated peptide (anti-CCP) 1170.8 units/mL (negative if <17 units/mL), while rheumatoid factor was negative at 3 (negative if <14 IU/mL). She first visited our rheumatology clinic for a second opinion approximately 18 months after her RA diagnosis. At that time, home medications included insulin, metformin, sitagliptin, indapamide, lisinopril, and rosuvastatin. She was subsequently treated with various conventional synthetic DMARD therapies, including sulfasalazine, hydroxychloroquine, and methotrexate (Table 1).

**Table 1:** History of disease-modifying anti-rheumatic drugs intake. Note: Month 0 represents the time of RA diagnosis. IV: intravenous; NSTEMI: non-ST elevation myocardial infarction; RA: rheumatoid arthritis

Medication	Dose	Period	Duration	Comments
Sulfasalazine	500 mg orally twice daily for one week, then 1000 mg twice daily	Month 0-Month 25	25 months	Discontinued to reduce polypharmacy
Hydroxychloroquine	200 mg orally twice daily	Month 0-Month 13	13 months	Self-discontinuation due to polypharmacy
Methotrexate	17.5 mg orally weekly	Month 14-present	Ongoing	94 months until time of submitting the case
Tocilizumab	8 mg/kg IV monthly	Month 25-Month 34	Nine months	Discontinued due to COVID-19 pandemic
Baricitinib	4 mg orally daily	Month 47-Month 80	≈33 months	NSTEMI at Month 79

To contextualize baseline and evolving CV risk, Table 2 summarizes blood pressure, pulse, BMI, renal function, glycemic control, lipid profile, inflammatory markers, and RA disease activity before, during, and after baricitinib therapy.

**Table 2:** Longitudinal cardiovascular risk, laboratory, and RA disease-activity parameters before, during, and after baricitinib therapy. Time points: Month 0 was defined as the time of RA diagnosis. T1 = at diagnosis, pre-treatment; T2 = at initiation of baricitinib add-on to methotrexate (Month 47); T3 = during sustained clinical remission on baricitinib and methotrexate (Month 47 to Month 80, approximately 33 months); T4 = at presentation with NSTEMI (Month 79); T5 = after discontinuation of baricitinib (Month 80 to Month 108, last clinic visit) on methotrexate monotherapy. Values shown as a single measurement or as a range across multiple measurements. anti-CCP: anti-cyclic citrullinated peptide antibody; bpm: beats per minute; dBP: diastolic blood pressure; CRP: C-reactive protein; DAS28-CRP: disease activity score in 28 joints based on CRP; NSTEMI: non-ST-elevation myocardial infarction; PGA: patient global assessment of disease activity; RA: rheumatoid arthritis; RF: rheumatoid factor; sBP: systolic blood pressure; VAS: visual analog scale

Parameter	Units	Reference range	T1 at diagnosis	T2 baricitinib initiation	T3 remission on baricitinib	T4 at NSTEMI	T5 post-baricitinib discontinuation
Blood pressure	mmHg	General target ≤130/80 in high-risk patients	sBP: 129; dBP: 63	sBP: 126; dBP: 78	sBP: 111-134; dBP: 69-80	sBP: 148; dBP: 74	sBP: 104-156; dBP: 51-79
Pulse	bpm	60-100	88	85	72-93	89	58-86
BMI	kg/m^2^	-	32.4	34.1	33.25-34.1	33.6	27.8-31.2
Serum creatinine	µmol/L	44-80	47	52	47-65	53	44-59
HbA1c	%	4.6-6.2	8.3%	10.8%	7.9-10.4%	10.4%	7.3-9.0%
Total cholesterol	mmol/L	3.9-5.2	3.86	3.55	3.34-4.49	3.57	2.70-4.15
HDL cholesterol	mmol/L	1.10-1.60	1.19	1.33	1.33-1.82	1.75	1.17-1.74
LDL cholesterol	mmol/L	≤2.59	2.17	1.55	1.24-2.27	1.24	1.02-1.96
Triglycerides	mmol/L	0.50-1.70	1.09	1.47	0.86-1.86	1.26	0.78–1.82
Total cholesterol/HDL cholesterol	-	≤4.50	3.24	2.67	2.04-2.74	2.04	2.05-3.01
Serum uric acid	µmol/L	143-339	210	156	150-270	190	152-210
CRP	mg/L	≤5.0	23.0	15.3	2.14-5.0	9.1	3.1-4.4
RA disease activity (DAS28-CRP)	Composite score	Target <2.6 (remission)	5.60 (high)	3.60 (moderate)	1.37-2.14 (remission)	2.14 (remission)	1.55-2.14 (remission)
PGA	mm (0-100 VAS)	Lower is better	95	60	0-10	40	0-20

She had chronic left shoulder pain due to osteoarthritis and rotator cuff disease. Around Month 18, she sought a second medical opinion outside the country and underwent a CV evaluation, including a dobutamine stress test and echocardiography, which reportedly did not show structural heart disease or inducible ischemia at that time. Due to persistent disease activity, intravenous tocilizumab was commenced around Month 25 and continued until around Month 34. It was then discontinued due to treatment interruption during the COVID-19 period, requiring a change in therapeutic strategy.

Given persistent disease activity on methotrexate and difficulty attending the hospital, baricitinib was added around Month 47. Her disease remained in remission for approximately 33 months, with a patient global assessment ranging from 0 to 10 mm, C-reactive protein (CRP) remaining within the normal range at 2.14 to 5.0 mg/L, and disease activity score in 28 joints (DAS28) - CRP ranging from 1.37 to 2.14. Approximately two weeks before the CV event at Month 79, her CRP increased to 9.1 mg/L, while her DAS28-CRP was 2.14, which still indicated remission with no synovitis. Her home medications included insulin, metformin, sitagliptin, indapamide, lisinopril, rosuvastatin, methotrexate, folic acid, baricitinib, and vitamin D.

At Month 79, while undergoing physiotherapy at our hospital for the left shoulder, the patient developed sudden-onset central chest pain radiating to the shoulder with rapid symptom progression, prompting urgent transfer to the emergency department. Electrocardiography revealed normal sinus rhythm and T-wave inversions in lead I, II, aVL, and V2-V6 (Figure 1), with a significant rise in cardiac troponin levels to 270 ng/L (normal range ≤ 14 ng/L), confirming acute NSTEMI. Transthoracic echocardiography was normal.

**Figure 1:**
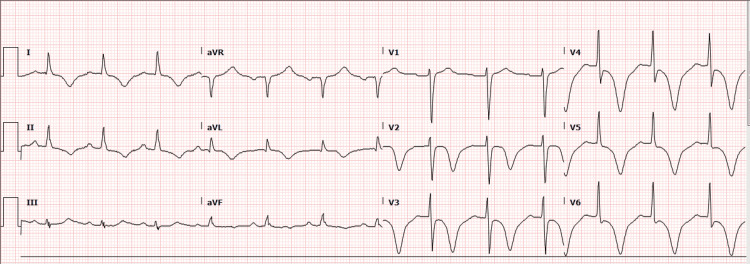
Electrocardiogram of the patient upon admission (Month 79), showing normal sinus rhythm with T-wave inversions in lead I, II, aVL, and V2-V6.

Rosuvastatin was replaced with high-dose atorvastatin daily. Aspirin was initiated, indapamide was replaced with bisoprolol, and temporary low-molecular-weight heparin was administered. Coronary angiography demonstrated severe calcified coronary artery disease, including 95% stenosis of the proximal to mid left anterior descending (LAD) artery and 95% stenosis of the ostio-proximal first diagonal branch. The patient underwent successful percutaneous coronary intervention (PCI) with revascularization of the LAD lesion. The patient continued taking methotrexate and baricitinib during the admission. She was discharged on dual antiplatelet therapy (aspirin and clopidogrel) for 12 months, followed by lifelong aspirin therapy. Metformin was replaced with empagliflozin/metformin. Other medications, including sitagliptin, vitamin D, calcium, insulin, methotrexate, folic acid, and baricitinib, were continued.

Following the CV event, the patient was reviewed in the rheumatology clinic at Month 80 when her RA was still in remission, and baricitinib was discontinued in light of the recent MI. Follow-up echocardiography at Month 97 demonstrated preserved left ventricular systolic function, with no regional wall motion abnormalities and grade I diastolic dysfunction, without additional significant structural abnormalities.

The patient has remained on methotrexate 17.5 mg orally once weekly as monotherapy for RA, and her disease remained in remission without further CV events until her last clinic visit (Month 108). At that visit, radiographs of the hands and feet, compared to radiographs taken 13 years earlier, showed stable periarticular osteopenia involving the carpal, metacarpophalangeal, and proximal interphalangeal joints, as well as osteoarthritic changes in some joints of her feet (Figure 2).

**Figure 2:**
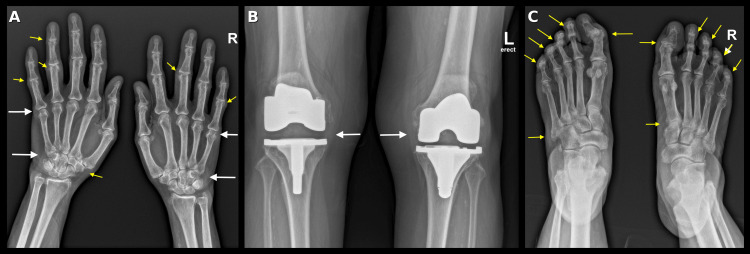
Radiographs of the hands (A), knees (B), and feet (C) show stable periarticular osteopenia involving the carpal and metacarpophalangeal joints (thick white arrows), with chronic osteoarthritic changes in several interphalangeal joints (yellow arrows). Knee radiographs show bilateral total knee replacements in good alignment (thick white arrows). Foot radiographs show osteoarthritic changes involving several joints of the feet (yellow arrows).

Knee radiographs, compared to radiographs taken eight years earlier, showed bilateral total knee replacements in good alignment. Bone densitometry of the lumbar spine and bilateral femoral necks, performed on a GE Lunar machine (GE Healthcare, Madison, Wisconsin, USA) at Month 94, was normal. She received intravenous zoledronic acid at approximately Month 51, Month 88, and Month 103.

Patient's perspective

The patient felt that baricitinib had been very effective in controlling her RA and had improved her daily life, especially because it was easier than attending the hospital for intravenous treatment. Because her arthritis had remained stable for a long period, she believed that her condition was under good control and did not anticipate a heart problem. She described the MI as sudden, frightening, and unexpected, especially since she had never experienced angina before and had previously been told that her cardiac evaluation was reassuring.

## Discussion

Patients with RA have a 69% increased risk of arterial ischemic CV events such as MI and stroke relative to the general population [2]. This is largely attributed to chronic systemic inflammation. In our case, the patient had a cardiac evaluation five years before the event that did not show inducible ischemia or structural heart disease at that time. However, these findings cannot exclude subsequent progression of coronary atherosclerosis. The patient developed NSTEMI after approximately 33 months of sustained clinical remission on baricitinib and methotrexate.

Available CV risk prediction tools may under- or overestimate risk in patients with rheumatic diseases [1]. The 2009 European Alliance of Associations for Rheumatology (EULAR) recommendations proposed adapting the Systematic Coronary Risk Evaluation (SCORE) by applying a 1.5 multiplication factor in patients with RA who meet at least two of the following three criteria: disease duration >10 years, RF or anti-CCP positivity, and certain extra-articular manifestations [9]. In our patient, this modified EULAR-SCORE approach was not applicable because she was older than the validated age range, had disease duration <10 years, and had no extra-articular disease. Using QRISK3, her estimated lifetime CV risk before MI was 66%. However, QRISK models for lifetime fatal and non-fatal CV risk were developed in the UK and incorporate the Townsend deprivation score, which may limit transportability to other populations [10]. The American Heart Association Predicting Risk of Cardiovascular Disease EVENTs (PREVENT) equations estimate 10-year and 30-year CV risk in adults without established CV disease. Before baricitinib initiation, our patient had no documented history of established atherosclerotic CV disease, including prior MI, stroke, transient ischemic attack, peripheral arterial disease, angina, or coronary revascularization. In a recent Spanish study, QRISK3 overestimated risk in patients with RA, whereas PREVENT CV disease was the only scale that performed reliably [11]. Using PREVENT CV, our patient’s estimated 10-year risk of coronary heart disease was 5.4%, and her estimated 10-year risk of total CV disease was 19.9%. None of these tools has been specifically validated in Arab patients with RA.

The Naranjo adverse drug reaction probability scale suggested a possible association between baricitinib and myocardial infarction (score = 3; Table 3) [12].

**Table 3:** Assessment of Naranjo adverse drug reaction probability scale in our patient. CV: cardiovascular; CRP: C-reactive protein; ECG: electrocardiogram; JAK: Janus kinase; N/A: not applicable; NSTEMI: non-ST-elevation myocardial infarction. Naranjo scale: definite ≥9, probable 5-8, possible 1-4, doubtful ≤0. Adapted from Naranjo et al. [12], with permission from John Wiley and Sons.

#	Criterion	Naranjo score	Assessment in this case
1	Previous conclusive reports of this reaction	+1 yes/0 no	+1: Three NSTEMI cases during baricitinib reported
2	Adverse event appeared after drug administration	+2 yes/-1 no	+2: NSTEMI after 33 months of baricitinib
3	Reaction improved after drug discontinuation (dechallenge)	+1 yes/0 no	0: No further CV events over 30 months post-discontinuation
4	Reaction reappeared on re-administration (rechallenge)	+2 yes/-1 no	0: Not performed - clinically inappropriate given severity of index event
5	Alternative causes present	-1 yes/+2 no	-1: Multiple CV risk factors present
6	Reaction reappeared with placebo	+1 yes/-1 no	0: Not performed
7	Drug detected at toxic concentrations	+1 yes/0 no	0: Not measured
8	Reaction more severe with higher dose	+1 yes/0 no	0: Fixed standard dose (4 mg/day) used throughout; no dose titration
9	Similar reaction to same or related drug previously	+1 yes/0 no	0: No prior similar reaction documented
10	Adverse event confirmed by objective evidence	+1 yes/0 no	+1: NSTEMI confirmed by troponin elevation, ECG changes, and coronary angiography
Total Naranjo score		+3	Possible adverse drug reaction

Regarding the Naranjo dechallenge item, although no recurrent CV events occurred after baricitinib discontinuation, this item was not scored as positive because the patient also underwent PCI and received secondary CV prevention, and no rechallenge was performed. Therefore, the absence of recurrence cannot be confidently attributed to baricitinib discontinuation alone. Even if the dechallenge item were scored positively, the total Naranjo score would be 4, which remains within the “possible” adverse drug reaction category.

Emerging pharmacovigilance data further complicate the interpretation of CV risk associated with JAK inhibitors. RCTs, pharmacovigilance, and real-world safety data remain inconsistent, with some analyses suggesting no significant increase in major CV adverse events (MACE) [7,13-15]. For example, analyses from the World Health Organization pharmacovigilance database (VigiBase) identified thromboembolic safety signals without a consistent increase in MACE across 126,815 cases [7]. Publicly accessible VigiAccess data also show that cardiac disorders are reported among suspected adverse events for baricitinib [16]. Long-term extension data from 3,770 patients with active RA treated with baricitinib for up to nine years showed that MACE incidence remained low and stable [15]. Similarly, RCTs and meta-analyses have generally shown a neutral effect of JAK inhibitors on CV and thromboembolic risk compared with placebo [13], particularly in clinical trial populations with relatively lower baseline CV risk. A recent meta-analysis found no meaningful difference in MACE risk between JAK inhibitors and TNF inhibitors across immune-mediated inflammatory diseases [17]. In contrast, other post-marketing and real-world analyses have raised concern for increased CV risk [3,8,18,19]. In particular, FDA Adverse Event Reporting System (FAERS) studies have identified disproportionate reporting of ischemic heart disease, heart failure, arrhythmias, and thrombotic events, particularly with baricitinib [18]. These discrepancies likely reflect differences in study design, patient selection, baseline CV risk, and outcome ascertainment across evidence sources.

These bodies of evidence should not be interpreted as equivalent. RCTs and meta-analyses provide higher internal validity for estimating treatment-associated risk, but they may include selected populations and may under-represent elderly patients with multiple comorbidities and high baseline CV risk. Long-term extension studies provide useful safety follow-up but remain influenced by trial selection and survivor bias. Real-world observational studies improve generalizability but are vulnerable to confounding by indication and differences in baseline CV risk. In contrast, spontaneous reporting systems such as FAERS, VigiBase, and VigiAccess are primarily signal-generating tools. They are affected by under-reporting, duplicate reporting, stimulated reporting after regulatory warnings, incomplete clinical information, and absence of denominator data; therefore, they cannot establish incidence, comparative risk, or causality.

Beyond RA-related CV risk, baricitinib may contribute to cardiometabolic effects through modulation of the JAK-STAT signaling pathway [20]. This pathway is implicated in endothelial function, platelet activity, lipid metabolism, and thrombotic regulation. Baricitinib therapy has been associated with dose-dependent increases in both low-density lipoprotein (LDL) and high-density lipoprotein (HDL) cholesterol levels [21], which may reflect alterations in systemic lipid handling and potentially influence atherosclerotic risk profiles. In addition, JAK-STAT inhibition has been proposed to affect thrombo-inflammatory pathways through mechanisms that may contribute to thrombotic risk [15]. However, available evidence suggests that CV safety outcomes with JAK inhibitors vary across indications and study populations, largely influenced by baseline CV risk, disease characteristics, and trial design [22]. This inconsistency suggests that observed CV outcomes may reflect an interaction between drug-related biological effects and underlying patient risk rather than a uniform class effect.

The approximately 33-month interval between baricitinib initiation and NSTEMI does not support an immediate acute drug-induced mechanism. However, it does not exclude temporal plausibility in the context of JAK inhibitor CV safety concerns, which may involve longer-term interaction between treatment exposure, lipid changes, thrombo-inflammatory pathways, RA-related CV risk, and progression of underlying atherosclerosis. Therefore, this event is best interpreted as a temporally associated NSTEMI during long-term baricitinib therapy in a high-risk patient, rather than as proof of direct causation.

JAK inhibitors should be used with caution in patients aged ≥65 years or in those with established CV risk factors, including hypertension, diabetes mellitus, hyperlipidemia, current or past smoking, or a history of atherosclerotic CV disease, as highlighted in regulatory safety communications following post-marketing safety data [23]. However, optimal strategies for CV monitoring during baricitinib therapy are not well defined in current guidelines. Furthermore, the extent to which aggressive modification of CV risk mitigates the risk of MACE in this population remains uncertain and warrants further investigation.

Risk stratification is an important prerequisite for the safe use of baricitinib in patients with CV risk factors. The tool shown in Figure 3 categorizes patients according to non-modifiable risk factors, including age >65 years, male sex, prior MI, cerebrovascular accident or venous thromboembolism, and history of malignancy, as well as modifiable risk factors such as poorly controlled hypertension, poorly controlled diabetes mellitus, poorly controlled hyperlipidemia, combined oral contraceptive use, and smoking [24].

**Figure 3:**
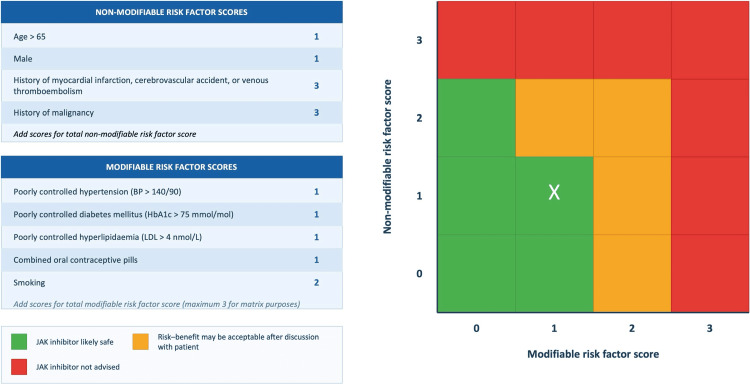
JAK Inhibitor Safety Decision Matrix for approximate risk stratification in patients with rheumatoid arthritis. JAK: Janus kinase; BP: blood pressure; HbA1c: glycated hemoglobin; LDL: low-density lipoprotein Reproduced from Harrington R, Harkins P, Conway R [24], under the terms of the Creative Commons Attribution 4.0 International License (CC BY 4.0).

Patients are then stratified into green, yellow, and red zones, reflecting likely safe use, cases requiring individualized risk-benefit discussion, and situations in which JAK inhibitor use is not advised. In our patient, age >65 years and poorly controlled diabetes mellitus would place her in the yellow zone, indicating that baricitinib should be prescribed with caution after discussion with the patient. Earlier application of this framework may have influenced the decision to initiate baricitinib in this patient. Ideally, implementation of structured risk stratification in routine clinical practice would prompt consideration of biologic agents with more established CV safety profiles prior to initiating JAK inhibitor therapy.

According to current regulatory guidance from agencies such as the European Medicines Agency (EMA) and FDA, JAK inhibitors should generally be reserved for patients in this category only when no suitable alternative therapies exist [25,26]. In this case, baricitinib was commenced due to persistent disease activity and limited therapeutic options, reflecting a clinically necessary but high-risk decision. Although dose reduction strategies (e.g., 2 mg once daily) have been proposed to mitigate MACE [25], their clinical impact remains uncertain. In our case, the patient received standard dosing and subsequently developed NSTEMI.

We searched MEDLINE/PubMed, Scopus, EMBASE, and Google Scholar for relevant literature up to 17 April 2026. The search terms included “baricitinib,” “rheumatoid arthritis,” “myocardial infarction,” “non-ST-elevation myocardial infarction,” “acute coronary syndrome,” “cardiac events,” and “major adverse cardiovascular events” (see Appendices). No restrictions were applied regarding study design or language. Retrieved records were screened by title and abstract, followed by full-text review of potentially relevant articles. The reference lists of included articles were also reviewed manually to identify additional relevant publications. Our comprehensive literature review identified three published cases. The first was reported in an RCT and involved a 46-year-old patient with RA, diabetes mellitus, and hypertension who developed NSTEMI on day 92 of baricitinib 4 mg daily [14]. No further details were provided. The second was described in real-world data from Spain and involved a 60-year-old woman with three CV risk factors, namely smoking, HDL cholesterol <40 mg/dL, and an extra-articular manifestation, who developed acute MI after 2.06 years of baricitinib 4 mg/day. Again, no further details were available [27]. The third was published as an abstract describing NSTEMI during baricitinib therapy in RA [28]. The patient continued baricitinib after the CV event, but the report did not provide essential information such as age, sex, dose, treatment duration, time to event onset, or outcome, limiting comparison with our case. Collectively, these reports provide only limited comparative value because key clinical details were lacking in all three.

Limitations of this study include its single-case design. Additionally, although the Naranjo scale was used as a structured pharmacovigilance aid, it should not be interpreted as proof of causality. Rather, this case represents a temporally associated and clinically well-documented NSTEMI occurring during long-term baricitinib therapy, with detailed longitudinal rheumatology follow-up, CV assessment, baricitinib discontinuation, and absence of recurrent CV events after dechallenge.

The value of the case lies instead in the real-world clinical scenario it represents: an elderly patient with RA, sustained inflammatory disease control, multiple CV risk factors, previous reassuring cardiac testing, and long-term exposure to a JAK inhibitor who developed an acute coronary event. This distinction is important because current safety concerns around JAK inhibitors are most relevant not to isolated drug causality, but to how clinicians identify, counsel, monitor, and re-evaluate patients whose baseline CV risk may evolve during treatment. Our case highlights the gap between regulatory recommendations and real-world clinical practice and reinforces the importance of individualized CV risk assessment when selecting JAK inhibitor therapy and determining the intensity of follow-up. The case also underscores the importance of reporting adverse drug reactions to hospital pharmacy departments and national pharmacovigilance authorities to strengthen post-marketing drug safety surveillance. Further real-world data from the Arab region are needed.

## Conclusions

This case provides detailed real-world documentation of NSTEMI occurring during long-term baricitinib therapy in an elderly patient with RA and multiple CV risk factors, including traditional risk factors and RA-related inflammatory risk, while highlighting a possible association in a high-risk clinical context rather than a causal relationship. It highlights the practical challenge of CV risk assessment during JAK inhibitor therapy in routine rheumatology care, where effective disease control, patient preference, and evolving CV risk must be balanced. Importantly, sustained inflammatory remission does not eliminate CV risk, particularly in patients with persistent or changing traditional risk factors. Therefore, this case underscores the need for periodic, structured CV reassessment, shared decision-making, careful patient selection, and pharmacovigilance during JAK inhibitor therapy, especially in high-risk patients and populations for which CV risk tools are not regionally validated. Further real-world registries from the Arab region are needed.

## Appendices

Literature search strategy

MEDLINE/PubMed Search Strategy

("Arthritis, Rheumatoid"[Mesh] OR "rheumatoid arthritis"[tiab])

AND

("Baricitinib"[Mesh] OR baricitinib[tiab])

AND

(

  "Myocardial Infarction"[Mesh]

  OR "myocardial infarction"[tiab]

  OR "acute myocardial infarction"[tiab]

  OR NSTEMI[tiab]

  OR STEMI[tiab]

  OR "acute coronary syndrome"[tiab]

  OR "coronary syndrome"[tiab]

  OR "ischemic heart disease"[tiab]

  OR "ischaemic heart disease"[tiab]

  OR "cardiovascular event*"[tiab]

  OR "cardiac event*"[tiab]

  OR "major adverse cardiovascular event*"[tiab]

  OR MACE[tiab]

)

Scopus Search Strategy

TITLE-ABS-KEY ( "rheumatoid arthritis" OR RA ) AND TITLE-ABS-KEY ( baricitinib ) AND TITLE-ABS-KEY ( "myocardial infarction" OR "acute myocardial infarction" OR MI OR NSTEMI OR STEMI OR "acute coronary syndrome" OR "coronary syndrome" OR "ischemic heart disease" OR "cardiovascular event*" OR "cardiac event*" OR "major adverse cardiovascular event*" OR MACE )

EMBASE Search Strategy

('rheumatoid arthritis'/exp OR 'rheumatoid arthritis':ti,ab OR ra:ti,ab) AND ('baricitinib'/exp OR baricitinib:ti,ab) AND ('myocardial infarction'/exp OR 'myocardial infarction':ti,ab OR 'acute myocardial infarction':ti,ab OR mi:ti,ab OR nstemi:ti,ab OR stemi:ti,ab OR 'acute coronary syndrome'/exp OR 'acute coronary syndrome':ti,ab OR 'coronary syndrome':ti,ab OR 'ischemic heart disease'/exp OR 'ischemic heart disease':ti,ab OR 'cardiovascular event*':ti,ab OR 'cardiac event*':ti,ab OR 'major adverse cardiovascular event*':ti,ab OR mace:ti,ab)

## References

[1] Gasparyan AY (2023). Cardiovascular manifestations and comorbidities in rheumatic diseases: perspectives on timely diagnosis, prevention, and treatment. Clin Rheumatol.

[2] Taylor PC, Weinblatt ME, Burmester GR (2019). Cardiovascular safety during treatment with baricitinib in rheumatoid arthritis. Arthritis Rheumatol.

[3] Goldman A, Galper BL, Druyan A (2024). Adverse cardiovascular events in rheumatoid arthritis patients treated with JAK inhibitors: an analysis of postmarketing spontaneous safety reports. Semin Arthritis Rheum.

[4] (2026). European Medicines Agency. Olumiant. https://www.ema.europa.eu/en/medicines/human/EPAR/olumiant.

[5] Mayence A, Vanden Eynde JJ (2019). Baricitinib: a 2018 novel FDA-approved small molecule inhibiting Janus kinases. Pharmaceuticals (Basel).

[6] Zavoriti A, Miossec P (2025). Understanding cardiovascular events with JAK inhibitors: similarities and differences of the vascular effects between different JAK inhibitors on endothelial cells exposed to inflammatory cytokines. ACR Open Rheumatol.

[7] Hoisnard L, Lebrun-Vignes B, Maury S (2022). Adverse events associated with JAK inhibitors in 126,815 reports from the WHO pharmacovigilance database. Sci Rep.

[8] Zhong X, Luo J, Huang Y, Wang S, Huang Y (2025). Cardiovascular safety of Janus kinase inhibitors: a pharmacovigilance study from 2012-2023. PLoS One.

[9] Agca R, Heslinga SC, Rollefstad S (2017). EULAR recommendations for cardiovascular disease risk management in patients with rheumatoid arthritis and other forms of inflammatory joint disorders: 2015/2016 update. Ann Rheum Dis.

[10] Hippisley-Cox J, Coupland C, Brindle P (2017). Development and validation of QRISK3 risk prediction algorithms to estimate future risk of cardiovascular disease: prospective cohort study. BMJ.

[11] Llorca J, Castañeda S, Ferraz-Amaro I (2025). Performance of four cardiovascular risk scales in chronic inflammatory rheumatic disease patients: 10-year follow-up data. Rheumatology (Oxford).

[12] Naranjo CA, Busto U, Sellers EM (1981). A method for estimating the probability of adverse drug reactions. Clin Pharmacol Ther.

[13] Campanaro F, Zaffaroni A, Cacioppo E (2023). Venous and arterial thromboembolic risk of Janus kinase inhibitors: a systematic review with meta-analysis. Rheumatology (Oxford).

[14] Genovese MC, Kremer J, Zamani O (2016). Baricitinib in patients with refractory rheumatoid arthritis. N Engl J Med.

[15] Taylor PC, Takeuchi T, Burmester GR (2022). Safety of baricitinib for the treatment of rheumatoid arthritis over a median of 4.6 and up to 9.3 years of treatment: final results from long-term extension study and integrated database. Ann Rheum Dis.

[16] (2026). World Health Organization. VigiAccess. https://www.vigiaccess.org/.

[17] Solitano V, Ahuja D, Lee HH (2025). Comparative safety of JAK inhibitors vs TNF antagonists in immune-mediated inflammatory diseases: a systematic review and meta-analysis. JAMA Netw Open.

[18] Mikaeili B, Alqahtani ZA, Hincapie AL, Guo JJ (2025). Safety of Janus kinase inhibitors in rheumatoid arthritis: a disproportionality analysis using FAERS database from 2012 to 2022. Clin Rheumatol.

[19] Yoon J, Kim S, Yang BR (2026). Cardiovascular adverse events of Jak versus TNF inhibitors using the Korean Pharmacovigilance database. Cardiology.

[20] O'Shea JJ, Plenge R (2012). JAK and STAT signaling molecules in immunoregulation and immune-mediated disease. Immunity.

[21] Qiu C, Zhao X, She L (2019). Baricitinib induces LDL-C and HDL-C increases in rheumatoid arthritis: a meta-analysis of randomized controlled trials. Lipids Health Dis.

[22] Bieber T, Feist E, Irvine AD (2022). A review of safety outcomes from clinical trials of baricitinib in rheumatology, dermatology and COVID-19. Adv Ther.

[23] Mongin D, Aymon R, Choquette D (2026). Evolution of Janus Kinase inhibitors (JAKi) prescriptions since 2015 in an international collaboration of rheumatoid arthritis registers (the 'JAK-pot' study): effect of regulatory warnings. Semin Arthritis Rheum.

[24] Harrington R, Harkins P, Conway R (2023). Janus kinase inhibitors in rheumatoid arthritis: an update on the efficacy and safety of tofacitinib, baricitinib and upadacitinib. J Clin Med.

[25] (2026). EMA recommends measures to minimise risk of serious side effects with Janus kinase inhibitors for chronic inflammatory disorders. Amsterdam: European Medicines Agency.

[26] (2026). FDA requires warnings about increased risk of serious heart-related events, cancer, blood clots, and death for JAK inhibitors that treat certain chronic inflammatory conditions. Food and Drug Administration.

[27] Rosas J, Belzunegui J, Hernández-Cruz B, Aguirregabiria I, Moyano S, Cobo A, Díaz-Cerezo S (2025). Real-world evidence for baricitinib in the treatment of rheumatoid arthritis in Spain: a systematic literature review. Adv Ther.

[28] Fitton J, Di Matteo A, Bissell LA (2025). Retrospective analysis of cardiovascular and malignancy risk factors in real world JAK inhibitor cohort and the effects of UK regulatory agency guidance cautioning JAK inhibitor use on prescribing. Ann Rheum Dis.

